# Controllable Hydrothermal Synthesis of 1D β-Ga_2_O_3_ for Solar-Blind Ultraviolet Photodetection

**DOI:** 10.3390/nano15050402

**Published:** 2025-03-06

**Authors:** Lingfeng Mao, Xiaoxuan Wang, Chaoyang Huang, Yi Ma, Feifei Qin, Wendong Lu, Gangyi Zhu, Zengliang Shi, Qiannan Cui, Chunxiang Xu

**Affiliations:** 1 State Key Laboratory of Digital Medical Engineering, School of Electronic Science and Engineering, Southeast University, Nanjing 210096, China; 220246876@seu.edu.cn (L.M.); 220212173@seu.edu.cn (C.H.); mayi@seu.edu.cn (Y.M.); lwd@seu.edu.cn (W.L.); zlshi@seu.edu.cn (Z.S.); qiannan@seu.edu.cn (Q.C.); 2 College of Telecommunications and Information Engineering, Nanjing University of Posts and Telecommunications, Nanjing 210003, China; qinfeifei@njupt.edu.cn (F.Q.); zhugangyi@njupt.edu.cn (G.Z.)

**Keywords:** hydrothermal, controllable synthesis, 1D β-Ga_2_O_3_ nanorods, solar-blind ultraviolet, photodetector

## Abstract

Gallium oxide (Ga_2_O_3_), an ultrawide bandgap semiconductor, is an ideal material for solar-blind photodetectors, but challenges such as low responsivity and response speed persist. In this paper, one-dimensional (1D) Ga_2_O_3_ nanorods were designed to achieve high photodetection performance due to their effective light absorption and light field confinement. Through modulating source concentration, pH value, temperature, and reaction time, 1D β-Ga_2_O_3_ nanorods were controllably fabricated using a cost-effective hydrothermal method, followed by post-annealing. The nanorods had a diameter of ~500 nm, length from 0.5 to 3 μm, and structure from nanorods to spindles, indicating that different β-Ga_2_O_3_ nanorods can be utilized controllably through tuning reaction parameters. The 1D β-Ga_2_O_3_ nanorods with a high length-to-diameter ratio were chosen to construct metal-semiconductor-metal type photodetectors. These devices exhibited a high responsivity of 8.0 × 10^−4^ A/W and detectivity of 4.58 × 10^9^ Jones under 254 nm light irradiation. The findings highlighted the potential of 1D Ga_2_O_3_ nanostructures for high-performance solar-blind ultraviolet photodetectors, paving the way for future integrable deep ultraviolet optoelectronic devices.

## 1. Introduction

Gallium Oxide (Ga_2_O_3_) has an ultrawide bandgap (larger than 4.6 eV), as well as high chemical and thermal stability. Hence, it has been applied in deep ultraviolet optoelectronic devices [1,2,3,4], particularly solar-blind ultraviolet photodetectors (SBPDs) [5,6,7]. Despite advancements in plane-structured Ga_2_O_3_ photodetectors, the limited light-sensitive area in them often leads to reduced light absorption efficiency, thereby affecting overall device performance. However, one-dimensional (1D) structural Ga_2_O_3_ offers distinct advantages, including high aspect ratio, enhanced light field confinement, and short electron transport paths. These properties contribute to improving optical absorption and energy harvesting, making 1D Ga_2_O_3_-based micro/nano structures promising for SBPD applications [8,9,10].

Until now, various methods have been undertaken to grow Ga_2_O_3_ nanostructures, such as chemical vapor deposition (CVD), chemical bath deposition, etc. For example, Fang et al. [11] utilized Ga_2_O_3_ microwires synthesized by the CVD method to construct a high-performance, self-powered, solar-blind violet external photodetector, and Wang et al. [8] further enhanced its current response by the piezoelectric effect. Moreover, in recent years, Shan et al. [12] fabricated the Sn-doped Ga_2_O_3_ microwires and realized high performance solar-blind ultraviolet photodetector. Li et al. [13] synthesized Ga_2_O_3_ nanobelts via a vapor-solid growth mechanism, and fabricated a high-sensitivity ultraviolet photodetector. However, the controllable synthesis and morphology tuning are difficult to achieve. Hydrothermal synthesis, on the other hand, offers a simple, cost-effective, and controllable route for fabricating micro/nanomaterials.

In this work, a straightforward hydrothermal method was employed to synthesize 1D Ga_2_O_3_ nanorods with a high length-to-diameter ratio and desirable morphology. By flexible controlling of the chemical reaction parameters, such as source concentration, pH value, temperature, and reaction time, the morphology characteristics of precursor GaOOH were manipulated efficaciously and studied systematically. Then, further high-temperature annealing treatment was employed to convert GaOOH into β-Ga_2_O_3_ nanorods with porous surface structure, which can effectively improve photodetector (PD) performance. Moreover, metal-semiconductor-metal (MSM) type photodetectors were then fabricated using the 1D β-Ga_2_O_3_ nanorods, which had great performance. The results illustrated the potential of 1D porous β-Ga_2_O_3_ nanomaterials in high-performance, solar-blind, ultraviolet photodetectors and deep ultraviolet optoelectronic applications.

## 2. Experiment

### 2.1. Materials Growth and Device Construction

The hydrothermal method has the advantages of simple operation, low cost, and high yield [14,15,16,17]. By adjusting reaction parameters and optimizing the growth process, the flexible control over the morphology and structure of samples can be achieved. In recent years, the hydrothermal method has been utilized to prepare Ga_2_O_3_ nanomaterials [18,19,20]. In this work, the first step was uniformly dissolving Ga(NO_3_)_3_·xH_2_O (99.9% metals basis, Aladdin^TM^) and C_6_H_12_N_4_ (99.5% calc. to the dried substance, Aladdin^TM^) in deionized water under magnetic stirring. Next, the clear solution was obtained and conveyed to a closed reaction chamber. Then, the chemical reaction occurred under the temperature range of 100~150 °C for different times to obtain the precursor sample of GaOOH nanorods. The hydrothermal synthesis process of GaOOH nanorods included two stages of crystallization and growth of the hydrolyzed products of Ga^3+^. Therefore, the hydrolysis process of Ga^3+^ can be expressed in the following way [21]:

$$C_{6}H_{12}N_{4}+6H_{2}O\to 6HCHO+4NH_{3}$$

$${NH}_{3}+H_{2}O\to {NH}_{4}^{+}+{OH}^{-}$$

$${Ga}^{3+}+3{OH}^{-}\to GaOOH+H_{2}O$$

More importantly, in the hydrothermal reaction process, all of the experimental parameters have non-negligible influences on the sample morphology characteristics, including the source concentration, pH value, temperature, and growth time [22,23,24]. Consequently, it is significant to optimize the hydrothermal reaction and thermal annealing processes to modulate the morphological characteristics of the expected 1D β-Ga_2_O_3_ nanorod arrays.

**Figure 1:**
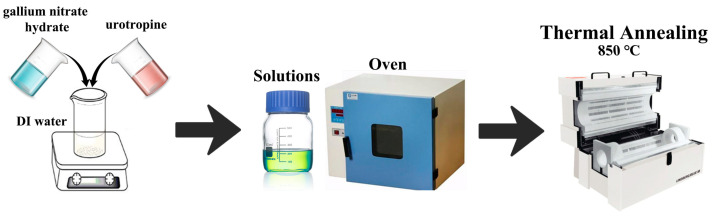
Schematic diagram of the synthesis process of the 1D porous β-Ga_2_O_3_ nanorods.

Next, the precursor samples further needed to be treated at high temperature (850 °C) after cooling naturally, washing by deionized water and ethanol, as well as drying. The high-temperature annealing treatment could transform the precursor to β-Ga_2_O_3_, and improve the crystallinity simultaneously. The entire synthesis process of 1D β-Ga_2_O_3_ nanorods is presented in Figure 1. The thermal decomposition process can be expressed by the following formula [23].

$$2GaOOH\underset{calcination}{\to }{Ga}_{2}O_{3}+H_{2}O$$

That is to say, the precursor GaOOH will transfer to the Ga_2_O_3_ by removing the formed water molecules under a high temperature. After obtaining the 1D porous β-Ga_2_O_3_ nanorods, the samples were homogeneously dispersed with anhydrous ethanol, and then slowly dripped and spun-coated onto the prepared Au interdigital electrodes with a width of 100 μm and space of 50 μm. Finally, the PD devices were obtained after drying.

### 2.2. Characterization Methods

The morphology of the nanorods was analyzed by field emission scanning electron microscopy (FESEM, Carl Zeiss Ultra Plus) equipped with an Energy dispersive spectroscopy (EDS) (Oxford X-Max 50). Their corresponding crystal properties were characterized with X-ray diffractometer (XRD) (Rigaku SmartLab) and Raman spectroscopy (Horiba France Sas XploRA). To obtain the bandgap of samples, the absorbance spectra were measured by an ultraviolet-visible (UV-vis) spectrophotometer (Shimadzu, UV-2600). In addition, the X-ray photoelectron (XPS, Thermo Scientific Nexsa) was employed to study the relevant chemical composition and valence states. The photoelectric properties of the photodetector were tested by a semiconductor parameter system (Keithley 4200), integrated with a light-emitting diode (LED) with a central wavelength of 254 nm as a light source.

## 3. Results and Discussion

### 3.1. Controllable Growth Process of GaOOH

**Figure 2:**
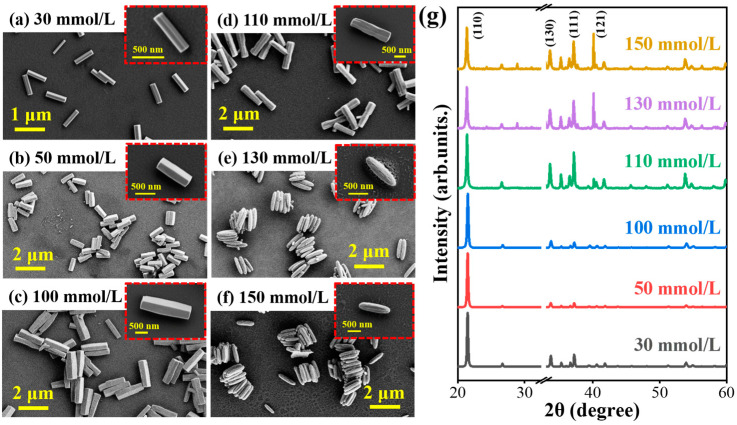
The influence of reaction source concentration on the produced GaOOH morphology: (**a**–**f**) SEM images and (**g**) XRD spectra of the samples fabricated with different Ga ion concentrations.

In order to study the precursor GaOOH growth process, the different reaction parameters in the hydrothermal method should be modulated, including the Ga ion source concentration, solution pH value, reaction temperature, and growth time. After that, the samples’ morphology and crystal characteristics should be compared and analyzed in detail. Firstly, with the same temperature (100 °C) and reaction time (100 min), the effect of Ga(NO_3_)_3_·xH_2_O concentration on the morphology and dimensions of GaOOH nanorods is studied by using various concentrations: 30 mmol/L, 50 mmol/L, 100 mmol/L, 110 mmol/L, 130 mmol/L, and 150 mmol/L. As presented in Figure 2, the scanning electron microscopy (SEM) images show noteworthy changes in both the length and morphology of the nanorods with increasing concentration. The GaOOH nanorods exhibit increased length with rising Ga ion concentration up to the 100 mmol/L, and the diameter of the nanorods increases slightly. In this process, the growth speed of the nanorods’ length accelerates with the increase of Ga ion source concentration. Further increasing the Ga ion concentration, the GaOOH nanorods show spindle-like morphologies emerged with a progressively rougher surface. This illustrates that the excess Ga ion source inhibits the nanorod growth and changes the crystal growth orientation.

In addition, the X-ray diffraction (XRD) patterns of the various samples are presented in Figure 2g to analyze the crystal characteristics of the GaOOH nanorods. When the reaction source concentration is lower than the 100 mmol/L, all the samples exhibit the similar crystal diffraction characteristics with several typical diffraction peaks. The diffraction peaks are related to the orthorhombic phase of GaOOH as identified by the reference code (JCPDS no.06-0180). In detail, the main three peaks located at 21.48°, 33.74°, and 37.22° are corresponding to the crystal plane (110), (130), and (111) of GaOOH, respectively. They are in concordance with the results obtained from the SEM images, indicating that GaOOH can manifest a rod-like morphology at relatively low concentrations. From the spectra, it is observed that the diffraction peak intensity ratio of the (110) and (130) crystal plane rises with the increase of the reaction source concentration up to 100 mmol/L. Additionally, a preferential orientation along the crystal plane (110) is clearly observed, identified as the polar plane of the GaOOH nanorods. This preferential orientation defines the synthesized 1D GaOOH nanorods. However, the ratio reduces continuously when the Ga ion source concentration exceeds 100 mmol/L. Moreover, a new strong diffraction peak corresponding to the (121) crystal plane also emerges in the XRD spectra shown in Figure 2g, indicating that the preferential growth orientation along the polar plane (110) is clearly weakened. Furthermore, the concurrent reduction in diffraction peak intensity corresponding to the (110) crystallographic plane accompanied by enhanced intensities from the (130), (111), and (121) planes, the changes both in the structure and surface are simultaneously obvious. The change of the crystal characteristics leads to a gradual transformation of the 1D rhombic nanorods to the irregular spindle-like shape. Notably, when the Ga ion concentration increases from 130 mmol/L to 150 mmol/L, the spindle-shaped nanorods exhibit only minor differences in both morphology and crystal characteristics, as illustrated in Figure 2. Hence, the Ga ion source concentration in the reaction solution has a crucial influence on the 1D GaOOH nanorod crystal growth, such as the polar surface growth rate, which can modulate the morphology. When the Ga ion source concentration is below the optimal value, the nanorods’ growth speed has a positive correlation with the source concentration. If the source concentration is higher than the optimal value, a different structure will be obtained, which results from the changed crystal growth speed of the different crystal plane. It is essential to select an appropriate Ga ion concentration for the synthesis of 1D GaOOH nanorods.

**Figure 3:**
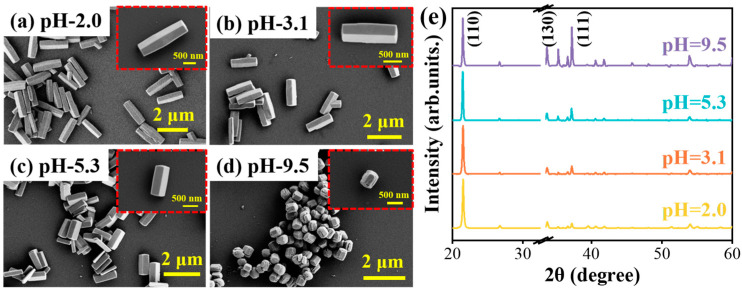
The influence of solution pH on the produced GaOOH morphology: (**a**–**d**) SEM images and (**e**) XRD spectra of the samples fabricated in different solution pH values.

Further, the solution pH value should be compared to study its influence on GaOOH nanorod morphology characteristics. Aiming to tune the pH value to 3.1, 5.3, and 9.5, the sodium hydroxide (NaOH) solution is added to the original mixed solution (pH 2.0) of the hydrothermal reaction process, as described above. The different samples are obtained by varying the solution pH values while keeping the other reaction parameters the same, including a temperature of 100 °C, time of 100 min, and Ga ion source concentration of the 100 mmol/L. As shown in Figure 3a–d, it is obvious that the nanorod length decreases gradually with an increase in the reaction solution pH. Moreover, the nanorods show the shortest length and splitting surface when the pH is up to 9.5. In addition, the corresponding XRD results shown in Figure 3e reveal that the diffraction peak intensity ratio between (110) and (111) reduces gradually with increases in solution pH values. Hence, the preferential growth speed along (110) becomes weaker. In the acidic environment with the low relative activity of hydroxyl (${OH}^{-}$) ions, the nanorods maintain preferential growth along the (110) crystal plane. On the contrary, under alkaline conditions, the growth rate along the polar plane (110) is inhibited. Notably, when the pH value increases beyond 11, there is barely any product in the reaction vessel. This suggests that excessive ${OH}^{-}$ ions hinder the formation of GaOOH nanorods, as they decompose under strongly alkaline conditions ($GaOOH+{OH}^{-}+H_{2}O\to {\left[Ga{\left(OH\right)}_{4}\right]}^{-}$). The different affinities of crystal facets to ${OH}^{-}$ ions are attributed to their anisotropic density of unsaturated bonds, leading to varying specific surface free energy densities [25,26,27]. Facets with higher densities of unsaturated bonds tend to absorb more ligands, promoting relatively faster growth speed. As a result, the mobility of ligands on distinct crystal facets primarily drives the morphological evolution of the nanorods under different pH conditions. The results demonstrate that the reaction pH condition can effectively modulate both the growth speed and the morphology of GaOOH nanorods.

**Figure 4:**
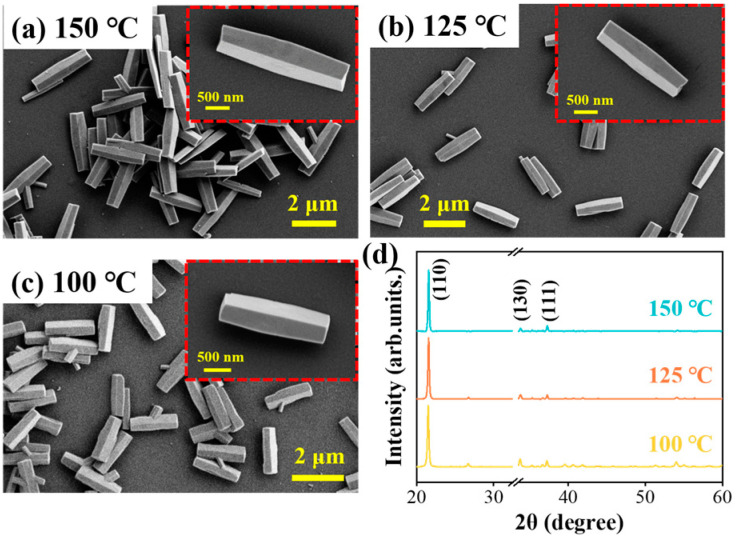
The influence of reaction temperature on the produced GaOOH morphology: (**a**–**c**) SEM images and (**d**) XRD spectra of the samples fabricated in different growth temperatures.

In addition, the reaction temperature is another crucial issue for the crystal growth process. To investigate the influence of reaction temperature on the precursor GaOOH nanorods, samples are synthesized at different temperatures while maintaining a constant reaction time of 100 min and Ga ion source concentration of the 100 mmol/L. From the SEM images in Figure 4a–c, the GaOOH nanorods keep the same structure, but exhibit an increased length with reaction temperatures increasing. Additionally, the XRD spectra in Figure 4d illustrate that all the samples present the same crystal structure and X-ray diffraction peaks. However, an increased reaction temperature leads to a higher peak intensity ratio between (110) and (130), indicating a faster growth rate along the polar plane (110). This result is consistent with the morphology changing that the nanorod length becomes longer, as demonstrated by the SEM images in the inset of Figure 4a–c. Consequently, the higher reaction temperature facilitates the synthesis of longer 1D GaOOH nanorods by improving the crystal growth rate of the polar plane (110).

**Figure 5:**
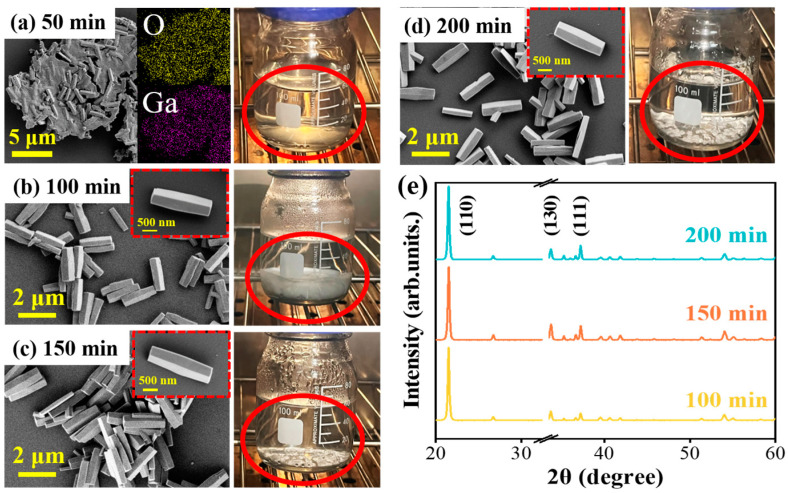
The influence of reaction time on the produced GaOOH morphology: (**a**–**d**) SEM images, elements distribution mappings, and snapshots of the samples in different times (50~200 min), and (**e**) XRD spectra of various samples. The red circles on the snapshots displays the precursor products in the reaction vessel.

Finally, the reaction time is the last parameter in the hydrothermal synthesis process of precursor GaOOH nanorods. To study the influence of growth time on the morphological characteristics of GaOOH nanorods, a series of samples are synthesized at varying reaction times while keeping other experiment parameters constant: reaction temperatures (100 °C), reaction source concentration (100 mmol/L), and solution pH value (2.0). The morphological and structural evolutions of the GaOOH nanorods at different stages are compared and analyzed. As shown in Figure 5a–d, the SEM images indicate that the obvious nanorod-like structures are embedded within the thin film in the early stage (50 min). According to the Energy Dispersive X-Ray Spectroscopy (EDX) measurements, the elements distribution mapping presented in Figure 5a confirms that both the thin film and nanorods are composed of Ga and O elements. Hence, the GaOOH nanorods start to form in this stage. As growth time increases, the nanorods become longer with more complete structure, while the thin film gradually disappears. After reaction times longer than 50 min, all the GaOOH nanorods grown by different times show a rhombic cross section, with diameters in the range of hundreds of nanometers and lengths extending to several micrometers. Consequently, the composite intermediate substance forms during the initial stage, from which the nanorods emerge and develop during the reaction process. As shown by the snapshots in Figure 5, the reaction solution emphasized with red circles transitions from clear to turbid in the transparent reaction flask, followed by the stratification and subsequent downward aggregation of white precipitates. The quantity of the samples increases with the reaction time increase. However, beyond 100 min, it starts to decline slightly by 150 min, but the structure of the products remains unchanged, as exemplified in the SEM image displayed in Figure 5c. This decrease in yield at extended reaction times is attributed to the common counter-dissolution process inherent to hydrothermal synthesis [28,29]. Further, the XRD spectra are measured and presented in Figure 5e. Based on the similar structure and dimensions, the samples display consistent XRD peak locations and intensities, which illustrate the similar crystal characteristics corresponding to 1D GaOOH nanorods. Additionally, these similar characteristics of samples from different reaction times indicate that GaOOH transforms into nanorods after the reaction time exceeds 50 min, and their properties remain if the reaction time is extended. Hence, the reaction time has barely any influence on the morphological characteristics of GaOOH nanorods, while it has obvious influence on the quantity of the products.

Above all, the hydrothermal method is proven to be an efficient and brief technology for the simple and controllable synthesis of 1D Ga_2_O_3_ nanostructure arrays. Through modulating the key parameters in the reaction process such as reaction time, temperature, source concentration, and solution pH, the morphology of nanostructures can be precisely tuned. These findings provide a solid foundation for further exploration in low dimensional optoelectronic devices. However, a systematic study of the subsequent thermal annealing process is necessary to obtain the different phased Ga_2_O_3_ nanorods derived from the precursor GaOOH nanorods.

### 3.2. Thermal Annealing GaOOH for β-Ga_2_O_3_

**Figure 6:**
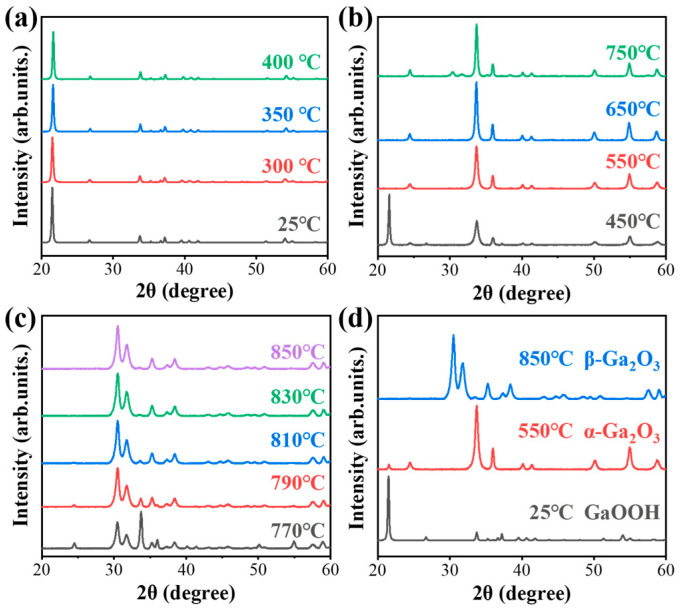
Thermal annealing in situ process: (**a**–**c**) XRD spectra of the same samples at different temperatures and (**d**) a comparison of different phases.

To investigate the effect of annealing temperature on the changes in nanorod crystal characteristics, XRD measurements were conducted in situ based on the sample (reaction temperature of 100 °C, Ga ion concentration of 100 mmol/L, and reaction time of 100 min). During the experiment, the samples were measured after annealing for 5 min at each temperature, and the results are presented in Figure 6. This approach enabled real-time observation of the transformation of crystal characteristics in GaOOH as the temperature increased. As shown in Figure 6a, the XRD patterns of the samples, annealed at temperatures ranging from 25 °C to 400 °C, present the diffraction peaks corresponding to the orthorhombic structure of GaOOH crystals, confirming that the nanorods remain in the original GaOOH phase within this temperature range. The nanorods maintain the same crystal characteristics, especially for the main diffraction peak (110), which corresponds to the polar plane of GaOOH. Moreover, the GaOOH nanorods always maintain the monocrystal characteristics in this temperature range. However, when the annealing temperature increases to 450 °C, the diffraction peak intensity ratio between the main peak of the crystal plane (110) at 21.49° and the other peaks obviously decreases. Furthermore, there are several new diffraction peaks appearing in the XRD spectrum, as displayed in Figure 6b, which is supposed to indicate a possible phase transformation of the nanorods.

As the annealing temperature reaches 550 °C, the disappeared diffraction peaks of GaOOH illustrate that the crystal phase transformation from GaOOH has been completed, and a new crystal phase has formed. Within the temperature range of 550~750 °C, the nanorods keep the same crystal phase, corresponding to the α-Ga_2_O_3_ (JCPDS no.06-0503). The main peaks appearing in the XRD are indexed as (104) at 33.72° and (110) at 35.98°, which show the higher-order diffraction peaks of the hexagonal structure of α-Ga_2_O_3_. Similarly, the crystal structure begins to transform to the monoclinic structure at 770 °C, as can be seen in Figure 6c. With the continuous increase in annealing temperature, the samples show the typical β-Ga_2_O_3_ crystal characteristics (JCPDS no.41-1103). The absence of any foreign phases confirms that the α-Ga_2_O_3_ was fully transformed into the monoclinic β-Ga_2_O_3_ phase following calcination at 850 °C. In addition, the relative intensities of (401), (002), (111), and (311) peaks, which are located at 30.49°, 31.82°, 35.28°, and 38.40°, increase in higher annealing temperature, indicating that the crystal orientation of these β-Ga_2_O_3_ crystals have better crystallinity as the annealing temperature increases. The XRD spectra in Figure 6d clearly illustrate that effective crystal phase transformation of the Ga_2_O_3_ nanorods can be achieved thorough annealing at specific temperatures. The transformation from GaOOH to α-Ga_2_O_3_ is completed at 550 °C, and this phase persists from 550 °C to 750 °C. By 850 °C, the transformation to β–Ga_2_O_3_ is complete with no foreign phases. Consequently, by selecting an appropriate thermal annealing temperature, a different phase of Ga_2_O_3_ nanorods can be obtained. Therefore, for achieving β-Ga_2_O_3_ nanorods, the temperature of 850 °C is chosen as thermal annealing temperature in subsequent experiments. Additionally, the size of the crystal plane in Figure 6d can be calculated with the Debye–Scherrer formula: $D=\frac{K\lambda }{\beta \cos\theta }$ [30,31,32], where λ is the X-ray wavelength of 1.54184 Å, θ is the diffraction angle, β is the FWHM of the diffraction peak, and K is a constant of 0.89. Firstly, the very sharp diffraction peak (110) at 25 °C illustrates the single crystal property for the GaOOH nanorods, where the Debye–Scherrer formula is not suitable for calculating the crystal size. After annealing treatment, the nanorods were transformed to polycrystal. Per the calculations, the crystal size of the α-Ga_2_O_3_ nanorods is 15.75 nm according to the diffraction peak (104), and the crystal size of β-Ga_2_O_3_ is 13.43 nm based on the peak (401). Moreover, the lattice parameters of GaOOH, α-Ga_2_O_3_, and β-Ga_2_O_3_ are calculated using XRD spectra information, which is indicated in Table 1. All lattice parameters in the table are consistent with the reference codes (GaOOH [JCPDS no.06-0180], α-Ga_2_O_3_ [JCPDS no.06-0503], and β-Ga_2_O_3_ [JCPDS no.41-1103]). Hence, the annealing treatment under different temperatures causes a similar polycrystal structure even with different phased Ga_2_O_3_.

**Figure 7:**
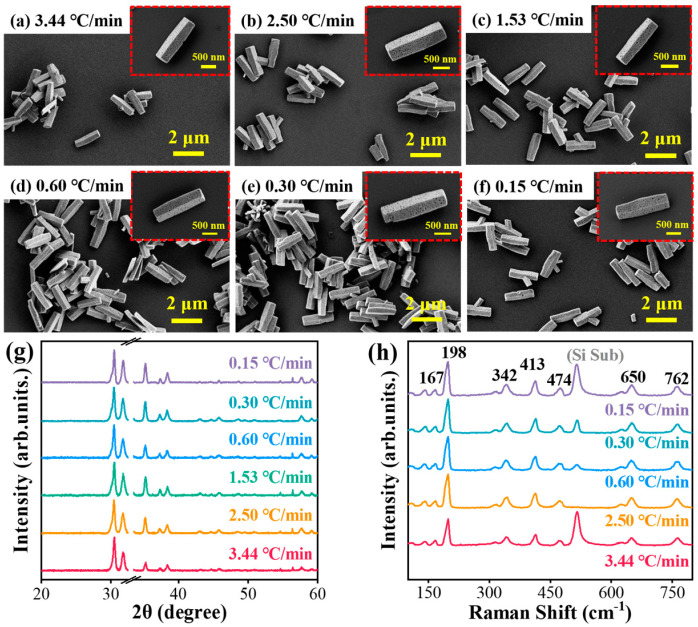
Thermal annealing speed influence on β-Ga_2_O_3_ nanorod: (**a**–**f**) SEM images and (**g**) XRD and (**h**) Raman spectra of the samples annealed at 850 °C with different rising temperature speeds.

Furthermore, the influence of the rising temperature rate on the nanorods is investigated with the thermal annealing temperature at 850 °C. As shown in Figure 7a–f, the SEM images reveal that the nanorods maintain nearly the same porous morphological characteristics after thermal annealing treatment. Moreover, the porous surface shows similar sizes and distributions even under the different rising temperature rates. The formation of this porous structure occurs via a dehydration reaction during the calcination process: $2GaOOH\underset{calcination}{\to }{Ga}_{2}O_{3}+H_{2}O$. However, it is important that 1D porous nanorods can enhance the light confinement ability, which is beneficial for further improving the photoelectronic response. In addition, the measured XRD peaks of the resulting β-Ga_2_O_3_ align well with the same monoclinic crystal structure (JCPDS no.41-1103), as displayed in Figure 7g. The dominant crystal plane diffraction peaks of (401), (002), and (111) are observed at 30.49°, 31.75°, and 35.28°, respectively. The locations and relative intensity ratio of these main peaks are almost identical, illustrating that the rising temperature rate has minimal impact on the morphological and crystal characteristics.

**Figure 8:**
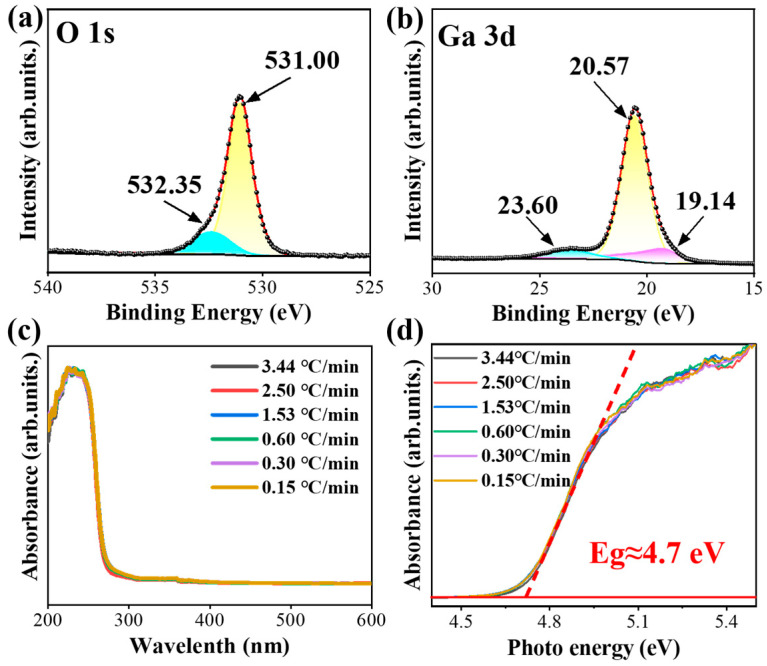
Thermal annealing speed influence on β-Ga_2_O_3_ nanorods: (**a**,**b**) high-resolution XPS spectra of O 1s and Ga 3d, and (**c**,**d**) normalized absorption spectra of the samples annealed at 850 °C with different rising temperature speeds.

Further characterization is conducted using Raman and X-ray photoelectron spectroscopy (XPS) measurements. The Raman spectra in Figure 7h show peaks respectively at 141 cm^−1^, 167 cm^−1^, and 198 cm^−1^, which correspond to low-frequency vibration modes and transitions within tetrahedron–octahedron chains [33]. The peaks located in the range of 300~500 cm^−1^ are related to the octahedral face of β-Ga_2_O_3_ (Ga_2_O_6_), while those between 600 cm^−1^ and 800 cm^−1^ correspond to the stretching vibrations of the tetrahedral face of β-Ga_2_O_3_ (GaO_4_) [34]. These characteristic peaks indicate that the nanorods exhibit a monoclinic crystal structure with C_2h_ group symmetry and high crystalline quality [35,36]. In addition, the XPS results provide valuable insight into the chemical composition and electronic binding states of the β-Ga_2_O_3_ nanorods. The high-resolution XPS spectra for Ga 3d and O 1s are depicted in Figure 8a,b. The O 1s peak in Figure 8a is deconvoluted into two peaks, including the first peak at 531.00 eV from the lattice oxygen in Ga_2_O_3_, and the peak at 532.35 eV from oxygen-deficient regions or chemical-adsorbed oxygen [37]. The high-resolution XPS spectrum of Ga 3d presented in Figure 8b is deconvoluted into three overlapping peaks at 19.14 eV, 20.57 eV, and 23.60 eV, which are attributed to the Ga-O bond [38]. These peaks are corresponding to the O^2-^ ions of the Ga-O bond and the O^2-^ in the oxygen-deficient regions, respectively. The detection of Ga and O elements on the surface, along with their corresponding binding energies, confirms that the powder calcined at 850 °C successfully crystallized into Ga_2_O_3_ with Ga and O atoms present with a stoichiometric ratio of 2:3. Moreover, Figure 8c,d presents the normalized optical absorption spectra of the β-Ga_2_O_3_ nanorods annealed at different rising temperature rates, which display a minimal difference. Furthermore, the bandgap of the β-Ga_2_O_3_ nanorods is about 4.7 eV. The ultra-wide bandgap of the β-Ga_2_O_3_ nanorods illustrates the main photoresponse in the solar-blind ultraviolet range.

### 3.3. Solar-Blind UV Photodetection

**Figure 9:**
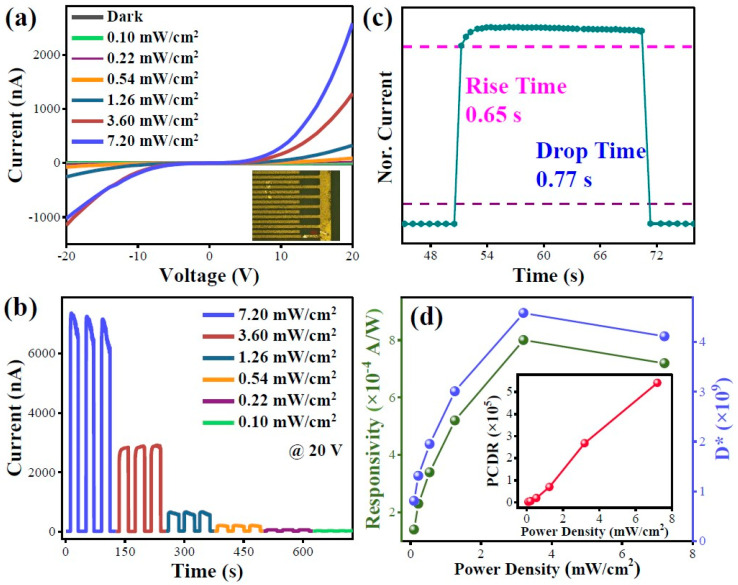
Photodetector parameters of 1D β-Ga_2_O_3_ nanorods: (**a**) I-V characteristics in the dark and under different power light irradiation, (**b**) I-T plots under different light power, (**c**) single periodic I-T plots irradiated under 3.6 mW/cm^2^, and (**d**) the relationship of PDCR, responsivity, and detectivity with light power density.

Based on the large length-to-diameter ratio and porous structure, the 1D β-Ga_2_O_3_ nanorods exhibit strong light absorption efficiency and optical confinement capabilities, which can improve the light absorption efficiency effectively to achieve high PD performance. To study the solar-blind ultraviolet photodetector performance based on the 1D β-Ga_2_O_3_ nanorods, the 1D porous nano-structural β-Ga_2_O_3_ (fabricated by 150 °C, 100 min, and 100 mmol/L Ga source concentration, and annealed for 2 h at 850 °C) is used to construct the MSM type device. The device presented in the inset of Figure 9a is created by spin-coating the nanorod samples onto the electrode substrate, followed by drying. Firstly, the I-V characteristics curves in Figure 9a illustrate that the photocurrent (*I_light_*) increases with increasing light power density. Under a 20 V bias, the dark current (*I_dark_*) is as low as 4.76 × 10^−12^ A, while *I_light_* reaches 2.58 × 10^−6^ A under the 254 nm light with an intensity of 7.2 mW/cm^2^, resulting in a high photo-to-dark current ratio (PDCR) of 10^6^. Furthermore, the periodic current-time (I-T) curves of the photodetector under different light power densities, depicted in Figure 9b, demonstrate that the current increases rapidly when the light is turned on and stabilizes, with the photocurrent gradually rising as the light intensity increases. Figure 9c presents the rise and decay time conducted on the photodetector at 20 V bias. The rise and fall times are defined as the times required for the photocurrent to increase from 10% to 90% or from 90% to 10% of the maximum value, and the measured typical rise and fall times of the device are 0.65s and 0.77s, respectively.

According to the equation, $R=\frac{I_{photo}-I_{dark}}{P\cdot A}$ and $D^{*}=R/\sqrt{2qI_{dark}A}$, the responsivity (R) and detectivity (D*) of the device can be calculated, where P is the optical power density, A is light spot area, and q is the elementary charge. The calculated results are summarized in Figure 9d. As the light intensity increases, R and D* of the device initially rise when the light power density is below 3.6 mW/cm^2^. At this intensity, R and D* reach their peak values of 8.0 × 10^−4^ A/W and 4.58 × 10^9^ Jones, respectively. However, further increasing the light power intensity to 7.2 mW/cm^2^ results in a decline in R and D*. This reduction is attributed to the depletion of distinct available states at high light power densities, as noted in previous studies [39,40]. These results highlight the high responsiveness and efficiency of the 1D porous β-Ga_2_O_3_ nanorods in detecting transient light signals. This exceptional performance underscores their potential as promising candidates for solar-blind ultraviolet photodetectors, which are crucial for a range of advanced optoelectronic applications.

## 4. Conclusions

In summary, the 1D porous Ga_2_O_3_ nanorods were synthesized using a straightforward and controllable hydrothermal method. By tuning reaction parameters in the growth process, such as the source concentration, pH value, temperature, and reaction time, the structure and morphology of the nanorods were flexibly modulated. The reaction temperature and source concentration in the low range had a positive correlation with the growth speed along the polar crystal planes of GaOOH nanorods. On the contrary, the excess Ga ion source and high pH conditions inhibited the growth process along the polar plane, changing the structure and morphology of GaOOH nanorods. Further, the thermal annealing treatment was optimized to obtain the expected 1D β-Ga_2_O_3_ nanorods. Based on the large length–diameter ratio, great light field confinement ability, and high field absorption efficiency, the MSM type photodetectors constructed with 1D β-Ga_2_O_3_ nanoarrays presented good deep ultraviolet photodetection performance. The responsivity of the photodetector reached 8.0 × 10^−4^ A/W, and the detectivity was as high as 4.58 × 10^9^ Jones. Although it was necessary to improve the photodetector performance for further optoelectronic devices and other applications, the results demonstrated excellent optical field absorption and confinement, and potential applications in micro/nano solar-blind photodetectors, which was significant for minimized and integrable optoelectronic devices in the deep ultraviolet range.

## Figures and Tables

**Table 1:** The lattice parameters of Ga_2_O_3_ nanorods in different phases.

	Structure	a (Å)	b (Å)	c (Å)	β(°)
GaOOH	orthorhombic	4.58	9.75	2.98	-
α-Ga_2_O_3_	hexagonal	4.99	-	13.49	-
β-Ga_2_O_3_	monoclinic	12.23	3.04	5.80	103.7

## Data Availability

Data is contained within the article.
